# Alternative nano-lithographic tools for shell-isolated nanoparticle enhanced Raman spectroscopy substrates†

**DOI:** 10.1039/d4nr00428k

**Published:** 2024-03-14

**Authors:** Ketki Srivastava, Thimo S. Jacobs, Stefan Ostendorp, Dirk Jonker, Floor A. Brzesowsky, Arturo Susarrey-Arce, Han Gardeniers, Gerhard Wilde, Bert M. Weckhuysen, Albert van den Berg, Ward van der Stam, Mathieu Odijk

**Affiliations:** a BIOS Lab on Chip Group, Mesa^+^ Institute of Nanotechnology, University of Twente The Netherlands m.odijk@utwente.nl; b Inorganic Chemistry and Catalysis group, Debye Institute for Nanomaterials Science and Institute for Sustainable and Circular Chemistry, Utrecht University The Netherlands w.vanderstam@uu.nl; c Institute of Materials Physics, University of Münster Germany; d Mesoscale Chemical Systems, Mesa^+^ Institute of Nanotechnology, University of Twente The Netherlands

## Abstract

Chemically synthesized metal nanoparticles (MNPs) have been widely used as surface-enhanced Raman spectroscopy (SERS) substrates for monitoring catalytic reactions. In some applications, however, the SERS MNPs, besides being plasmonically active, can also be catalytically active and result in Raman signals from undesired side products. The MNPs are typically insulated with a thin (∼3 nm), in principle pin-hole-free shell to prevent this. This approach, which is known as shell-isolated nanoparticle-enhanced Raman spectroscopy (SHINERS), offers many advantages, such as better thermal and chemical stability of the plasmonic nanoparticle. However, having both a high enhancement factor and ensuring that the shell is pin-hole-free is challenging because there is a trade-off between the two when considering the shell thickness. So far in the literature, shell insulation has been successfully applied only to chemically synthesized MNPs. In this work, we alternatively study different combinations of chemical synthesis (bottom-up) and lithographic (top-down) routes to obtain shell-isolated plasmonic nanostructures that offer chemical sensing capabilities. The three approaches we study in this work include (1) chemically synthesized MNPs + chemical shell, (2) lithographic substrate + chemical shell, and (3) lithographic substrate + atomic layer deposition (ALD) shell. We find that ALD allows us to fabricate controllable and reproducible pin-hole-free shells. We showcase the ability to fabricate lithographic SHINER substrates which report an enhancement factor of 7.5 × 10^3^ ± 17% for our gold nanodot substrates coated with a 2.8 nm aluminium oxide shell. Lastly, by introducing a gold etchant solution to our fabricated SHINER substrate, we verified that the shells fabricated with ALD are truly pin-hole-free.

## Abstract 2

This work shows increased stability and homogeneity for shell-isolated nanoparticle enhanced Raman spectroscopy substrates fabricated by using a combination of lithography and atomic layer deposition, compared to chemically synthesized substrates.

## Graphical abstract

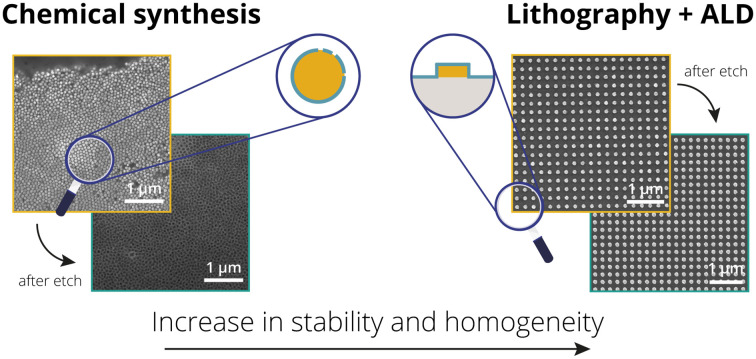

## 1. Introduction

Surface-enhanced Raman spectroscopy (SERS) has proven to be a remarkable tool to study and monitor chemical reactions.^1–3^ SERS has been applied to study electrochemical,^4^ plasmon-driven,^5^ and heterogeneous catalytic reactions.^2^ It relies on the resonant coupling of the incident electromagnetic radiation to the localized conduction electrons oscillating in the plasmonic metal nanoparticles (MNPs). This increases the local electric field generated between two neighbouring MNPs which appear as ‘hot spots’ in the electric field distribution at the boundaries of the MNPs.^6–8^ These nanoscopic hot spots offer high chemical sensitivity, enabling the observation and characterization of reactants and products at the single-molecule level.^9,10^ SERS can also be used to study chemical processes with an improved time resolution, as the signal-to-noise ratio is better than for conventional Raman spectroscopy.^11^ However, the plasmonic driven electric field divergence can polarize adsorbed surface species and drastically impact the ongoing chemical reaction, for example by influencing the analyte bonds.^12,13^ SERS substrates can also dissipate small amounts of heat upon plasmonic excitation, resulting in a change in the local analyte concentration or the overall temperature of the chemical reaction.^3^ Another possible outcome is the formation and injection of hot electrons, which can induce alternative pathways for the chemical reaction.^14^ Lastly, in the example above, we only consider cases where the metal nanoparticle acts as both a plasmon-enhancing substrate and a catalytic surface where the chemical reaction occurs.^15^ However, not all chemical reactions use plasmonic metal catalysts; therefore, such SERS substrates are not always ideal for all chemical reactions as most of the active metal particles for catalysis have weak plasmonic properties.

The concept of “borrowing” SERS activity was therefore introduced to overcome the limitation of always requiring a plasmonic metal. A metal nanoparticle substrate is essentially used as a plasmon-enhancing base, atop which catalytic particles are deposited or uniformly self-assembled.^16^ When the chemical reaction takes place at the catalyst surface, the products formed can be easily detected due to their presence in/near the high intensity electric field hot spots created between two neighbouring plasmonic nanoparticles. However, plasmonic materials, typically coinage metals such as silver (Ag), gold (Au) and copper (Cu), which are also in contact with the chemical reaction, may also potentially influence the studied chemical reaction. Therefore, an inert coating such as silicon dioxide (SiO_2_) or aluminum oxide (Al_2_O_3_) is required to prevent the participation of the plasmonic nanoparticle in the chemical reaction by lowering the adsorption of the chemical species to the surface of the plasmonic metal nanoparticle.^1,12,17^ Furthermore, the shell needs to be thin enough so that the decaying electric field still has a large enough magnitude to perturb the chemical environment.

This concept, known as shell-isolated nanoparticle-enhanced Raman spectroscopy (SHINERS), has emerged as a powerful tool for probing catalytic reactions.^18–20^ Next to the thickness of the shell being typically in the range of 2–5 nm, it is also imperative that the shell is durable, conformal, and stable under catalytic reaction conditions (for example, temperatures typically above 300–400 °C).^20,21^ Generally, the concept of SHINERS has, up until now, only been applied to chemically synthesized nanoparticles.^18,22,23^ Various metal oxides are used to prepare insulating shells on the plasmonic nanoparticles using chemical synthesis.^24^ For example, Au@SiO_2_ shell isolated nanoparticles (SHINs) were used to study the hydrogenation of *para*-nitrothiophenol (pNTP) on platinum-based catalysts.^25^ Furthermore, it has been shown in literature that SHINs such as Au@SiO_2_, and Au@TiO_2_ show the ability to study the hydrogenation of CO using ruthenium catalyst particles,^1^ the reduction of NO using rhodium catalyst particles^26^ as well as the hydrogenation of acetylene using nickel catalyst particles.^27^

For SHINs, the insulating shells must be pin-hole-free in order to prevent the interaction of the chemical species with the enhancing nanoparticles. The likelihood of pin-holes being present in a shell increases drastically as the shell is made thinner. The increased presence of exposed metal can lead to alternative and/or competing reactions to interfere with the desired chemical reaction, which is not always desirable.^28^ Achieving good control over the shell thickness and ensuring high enhancement despite the presence of a pin-hole-free shell is, therefore, of utmost importance in using SHINs for catalysis. To rule out the presence of pin-holes, different methods can be employed to evaluate the characteristics of the insulating shell. High-resolution transmission electron microscopy (HR-TEM) can be used to analyze the shell thickness and uniformity in a 2D plane. The interaction of pyridine with the plasmonic MNPs is often used to indicate the presence of pin-holes.^12^ As pyridine is only adsorbed on metal surfaces, a fully insulated MNP will not show the characteristic pyridine vibrations in the Raman spectrum (1008 and 1030 cm^−1^). Lastly, the absence of an Au reduction peak in H_2_SO_4_ solution during cyclic voltammetry experiments can also be used as an alternative method to confirm the presence of a pin-hole-free shell.^12^

Although significant advances have been made in the field to have good control over the shell thickness and uniformity, the chemical synthesis method still lacks reproducibility. The spatial variation in shell thickness results in a high variance of the signal strength of the SHINs. Another major contribution to the variance in the signal strength is the random distribution of the drop-casted SHINs on a substrate due to the coffee-stain effect.^29^ In addition, non-uniformity in the insulating shell also contributes to the poor reproducibility of SHINs.

To overcome the challenges regarding the use of inert coatings with consistent shell quality and thickness while maintaining a strong electric field, is vital for a reliable application of SHINs in catalysis. Therefore, in this work, we evaluate the SERS performance of three different approaches to fabricate SHINER substrates. These include (1) chemically synthesized MNPs + chemical shell, (2) lithographic substrate + chemical shell, and (3) lithographic substrate + atomic layer deposition (ALD) shell. Based on our studies, we firstly propose using lithographic SERS structures to replace chemically synthesized nanoparticles. Using lithographic tools for fabrication not only offers precise control over the structure's size, shape, and geometry but also provides extremely good control over the periodic arrangement of the enhancing structures. Secondly, to fabricate conformal and thin insulating shells (∼3 nm), we propose using ALD as an alternative method to chemically synthesized shells. Since ALD is a self-limiting, monolayer deposition technique, it offers great control over the thickness of the produced shell. We show that the combination of lithography with ALD offers reproducible shell-isolated SERS substrates. Lastly, to ensure a pinhole-free shell, we also introduce using Au etchant as an indicator for testing the stability and durability of the insulating shells. Compared to HR-TEM and pyridine signals, which might be limited due to equipment resolution, we find that the Au etchant test proved to be a reliable method for the detection of pin-holes. As a proof of concept, we show the ability to fabricate lithographic SHINERS consisting of a gold nanodot array substrate coated with a 2.8 nm aluminum oxide shell and report an enhancement factor of 7.5 × 10^3^ ± 17% for the same substrate.

## 2. Materials and methods

(3-Aminopropyl)trimethoxysilane (APTMS, 97%), sodium silicate solution (reagent grade), rhodamine 6G (95%), hydroxylamine hydrochloride (NH_2_OH HCl, >98%) from Sigma-Aldrich; ammonia solution (28–30%) from VWR International; HAuCl_4_·3H_2_O (99.99% metals basis) from Alfa Aesar; trisodium citrate dihydrate (99%) from Acros Organics; hydrochloric acid (Emsure, 37%) from Merck. Before use, demineralized water was purified with a Milli-Q system (18.2 MΩ). All chemicals were used as received.

### 2.1 Approach 1: Au and shell-isolated nanoparticle

The synthesis procedure was based on the work of T. Hartman *et al.*^1^

#### 2.1.1 Seed synthesis

Ultra-pure H_2_O (30 mL) and 1 wt% HAuCl_4_ (30 mL) were heated at boiling point in a 250 mL round-bottom flask with a clean stirring bar (stirring at 1000 rpm). Once the solution started boiling, a 1 wt% sodium citrate (1 mL) was added to the solution. The heating was removed after 15 min.

#### 2.1.2 Au nanoparticle synthesis

The as-prepared seeds (1 mL) were added to the ultra-pure H_2_O (112 mL) and 1 wt% sodium citrate (2 mL) was added. The nanoparticles were carefully grown by dropwise addition of NH_2_OH HCl (1.6 mL, 10 mM) and HAuCl_4_ (2 mL, 0.5 wt%) over 30 min. The solution was stirred for an additional 10 min after the particles were fully grown.

#### 2.1.3 Shell-isolated nanoparticle synthesis

A solution containing 2 mM (3-aminopropyl)trimethoxysilane (APMTS, 0.2 mL) was added dropwise to a solution of Au nanoparticles (15 mL). The solution was stirred for 15 min. A sodium silicate solution (1.8 mL, diluted to 0.54 wt% with ultra-pure H_2_O and adjusted to pH 9.8 with 1 M HCl) was added under vigorous stirring. The flask was heated to 80 °C and kept at that temperature for 20 min. Subsequently, the solution was cooled in a water/ice bath. The solution was then centrifuged (5 min @ 5000 rpm) and washed three times with ultra-pure H_2_O.

#### 2.1.4 Transmission electron microscopy imaging

Transmission electron microscopy (TEM) images were collected with a FEI Tecnai 20 electron microscope, using an acceleration voltage of 200 kV.

### 2.2 Approach 2: Au NP on silicon nanocone (AuNP@SiNC)

All fabrication was done at the Mesa^+^ Institute of Nanotechnology, University of Twente, The Netherlands. The shell deposition experiments were conducted at the Debye Institute for Nanomaterials Science, Utrecht University.

#### 2.2.1 Au nanoparticle on silicon nanocone fabrication

The Au nanoparticle on silicon nanocone (AuNP@SiNC) geometry was fabricated according to the methods described by Jonker *et al.*^30^ The SiNCs were fabricated using a combination of Displacement Talbot Lithography (EULITHA PhableR 100C) and reactive ion etching (OXFORD INSTRUMENTS PlasmaPro 100 Estrelas) The SiNCs are arranged in a square periodic lattice with a pitch of 250 nm. The deposition of 65 nm AuNP on the SiNC was performed using a discrete-rotation glancing angle deposition technique. The Au deposition was carried out in an electron beam evaporator (BALZERS BAK 600) with an acceleration voltage of 10 kV and a beam current of 280 mA. The optimized deposition rate of 0.1 nm s^−1^ was used for all depositions.

#### 2.2.2 Shell coating of Au nanoparticle on silicon nanocone (SiO_2_ shell – AuNP@SiNC)

A solution of 4 mM APTMS in 50 mL ultra-pure H_2_O was prepared in a volumetric flask. The solution was allowed to hydrolyze for a couple of minutes. The wafer containing the Au@SiNC geometry was placed in the solution using a reversed carbon tweezer, and the solution was stirred for 1 hour. Subsequently, the wafer was washed in ultra-pure H_2_O and dried with an air gun. A sodium silicate solution (1.4 mL, diluted to 0.27 wt% with ultra-pure H_2_O and adjusted to pH 9.8 with 1 M HCl) was mixed with 15 mL ultra-pure H_2_O in a flask and heated to 70 °C while stirring. The wafer was placed in the solution after the required temperature was reached and kept in the solution for 40 min, after which it was rinsed with ultra-pure H_2_O and dried with an air gun.

### 2.3 Approach 3: lithographic SERS substrate fabrication with atomic layer deposition shells

All fabrication was done at the Mesa^+^ Institute of Nanotechnology, University of Twente, The Netherlands. A detailed fabrication process flow of the Au nanodots and Au nanoholes can be found in the ESI.† The ALD of Al_2_O_3_ shells was carried out at the Institute of Materials Physics, University of Münster, Germany.

#### 2.3.1 Au nanodots

Au nanodots were fabricated using electron beam lithography (Raith EBPG5150) and metal deposition lift-off. A 255 nm photoresist (PMMA A4) was spun on a clean Si wafer (one-sided polished (OSP), 〈100〉) and baked at 180 °C for 90 s. For the exposure, a dose of 1100 μC m^−2^ with a beam current of 5 nA was used to write 100 nm dots with an array spacing of 120 nm. After development in 1 : 3 MIBK : IPA (methyl isobutyl ketone : isopropanol) solution for 90 s, 3 nm chromium (Cr) and 55 nm Au was sputtered (T'COathy) on the wafer. The wafer was then subjected to lift-off in acetone overnight, followed by sonication in DI water for 20 min. Prior to the fabrication, a dose test was also performed to determine the optimal dose for the fabrication of nanodots.

#### 2.3.2 Au nanoholes

Au nanoholes were fabricated using Displacement Talbot Lithography (DTL). Prior to the exposure, a silicon (OSP, 〈100〉) wafer was coated with bottom anti-reflection coating (BARC, Barli-II, AZ Microchemicals) which was spun at 3000 rpm for 45 s. After the spin coating, the wafer was baked at 185 °C to form a layer with a thickness of ∼200 nm. The wafer was then coated with a commonly used DTL photoresist (PFI-88, PGMEA 1 : 1, Sumitomo) at 4000 rpm for 45 s. The photoresist was then baked at 90 °C for 60 s, thereby forming a layer thickness of ∼300 nm. For the exposure, a 375 nm I-line laser was used and the photoresist along with the BARC were exposed twice to form an array of nanodots. The exposure was carried out using a DTL machine (EULITHA PlableR 100C) for 45 s at each exposure cycle. During the DTL process, a gap of 60 μm was maintained, and a laser power of 0.98 mW cm^−2^ was used. After the exposure, the wafer was baked again at 90 °C and then developed in a OPD4242 solution for 60 s followed by rinsing with DI water. Fig. S1† shows an image of the photoresist nanodots formed after development. Sputtering (T'COathy) was then used to deposit a 3 nm Cr film and 55 nm Au film. For the lift-off process, the substrate was submerged in 99% nitric acid (HNO_3_) for 15 s to dissolve and remove the BARC. This was done as BARC is soluble only in HNO_3_ and not in solvents such as acetone, isopropanol and ethanol. The wafer was finally subjected to sonication to remove residues and rinsed with ethanol and deionized (DI) water.

#### 2.3.3 Atomic layer deposition

Trimethylaluminum (TMA – STREM min 98%) and DI water were used as precursors to form controlled thin films of aluminum oxide (Al_2_O_3_) on various substrates. The ALD system (CAMBRIDGE NANOTECH Savannah 100) was operated with nitrogen as inert carrier gas either at 150 °C or 250 °C, resulting in different microstructures of the formed Al_2_O_3_. Prior to deposition, the system is thoroughly degassed/cleaned from previously used precursors and allowed to stabilize for 1 h. The base pressure of the ALD system at a steady 20 sccm flow of nitrogen is ∼1 Torr (133 Pa). In order to form a monolayer of Al_2_O_3_, firstly water (vapor) is injected into the carrier gas flow, reacting with all accessible surfaces in the ALD-system including the sample surface. After the surplus water precursor is removed from the system, TMA (vapor) is injected into the carrier gas flow, reacting with all hydroxy-terminated surfaces left by the water vapor. Removing the surplus TMA precursor completes one deposition cycle. The carrier gas flow, the precursor injection timing, and the vacuum supply are modified multiple times throughout this cycle process to allow a longer reaction time of the precursors with the surfaces without raising the process pressure too much to insecure levels. A detailed process outline is described in the ESI Table S1.† By repeating this cycle, well-defined layer thicknesses of Al_2_O_3_ can be achieved.

### 2.4 Raman measurements

The Raman spectra were measured using a HORIBA Raman Spectrometer with a 638 nm laser, 1200 mm^−1^ grating, 50× objective (numerical aperture, NA = 0.5), and 10% laser power (1.60 mW or 10^5^ W cm^−2^). An edge filter was used to filter out the excitation light. The Au nanoparticles and SHINs in solution were drop cast onto a microscopy slide (10 μL), and the liquid was evaporated in air. The Raman signal enhancement tests were performed by adding an aqueous Rhodamine 6G (Rh6G) (0.1 mM) solution over the drop-casted substrates and covering the sample with a glass cover. The pin hole tests were performed by adding an aqueous pyridine (10 mM) solution over the drop-casted substrates and covering with a glass cover. The glass covers were applied to prevent evaporation of the analyte solutions.

## 3. Results and discussion

In this work, three ways to fabricate SHINs with uniform enhancement were investigated. These are: (1) chemically synthesized MNPs + chemical shell, (2) lithographic substrate + chemical shell and (3) lithographic substrate + ALD shell. In the first approach, we evaluated the performance of traditional chemically synthesized SHINs. Here, the SERS substrate (consisting of drop-casted metal nanoparticles) and the insulating shells were chemically synthesized. In the second approach, instead of using a chemically synthesized SERS substrate base, we used a lithographically fabricated SERS substrate (AuNP@SiNC) and prepared the insulating shells *via* the classical chemical approach, similar to approach 1. Finally, in the third approach, we evaluated the performance of a SHINER substrate that consists of a lithographically fabricated SERS substrate base and an ALD-prepared insulating shell. Various characterization techniques were applied to evaluate the presence of pin-holes and the Raman enhancement capabilities of SHINER substrates in each case. To avoid confusion with the nomenclature, it should be noted that the SHINER substrate basically consists of a SERS substrate base and an insulating shell.

### 3.1 Approach 1: chemically synthesized Au nanoparticles and shell-isolated nanoparticles

**Fig. 1:**
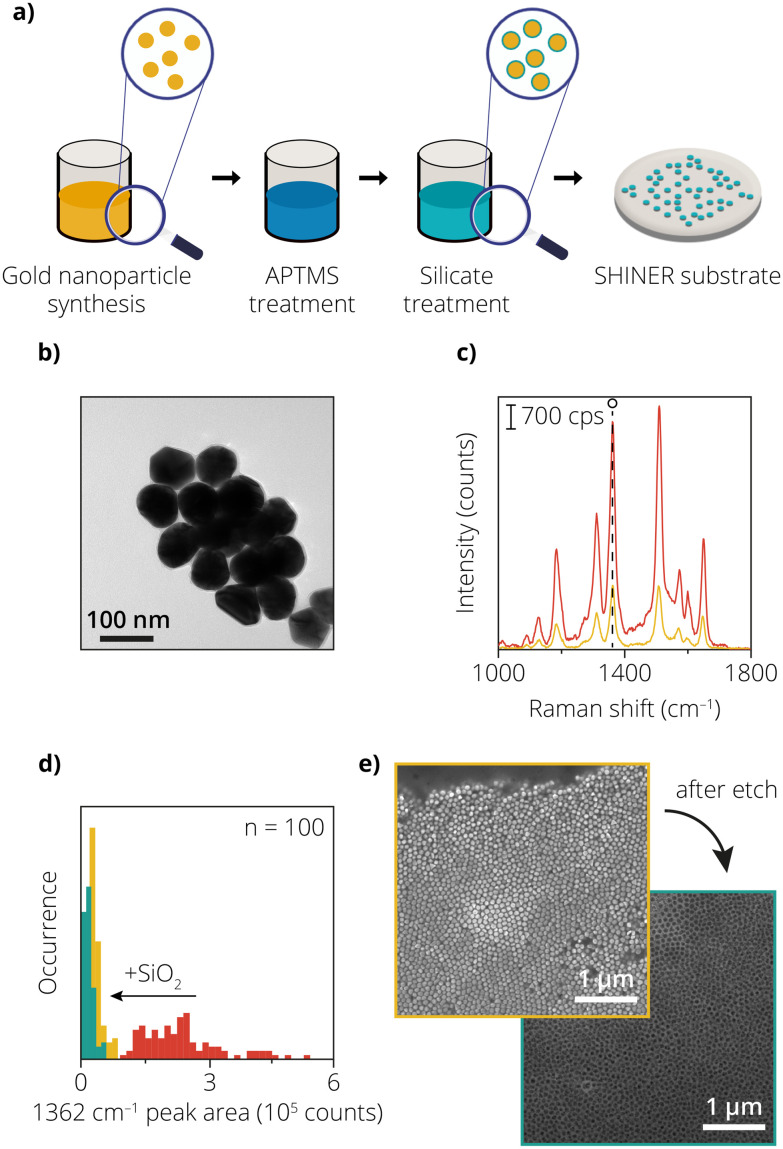
(a) Schematic overview of the preparation of the shell-isolated nanoparticles (SHINs), starting with synthesizing the Au nanoparticles and subsequently the silica coating with the APTMS/sodium silicate. (b) Transmission electron microscopy (TEM) image of the Au@SiO_2_ SHINs. (c) Raman spectrum of the bare Au nanoparticles (red) and Au@SiO_2_ SHINs (yellow) after adding the Rh6G solution, the 1362 cm^−1^ peak is marked with the open circle (o). (d) Histogram of the peak areas of the 1362 cm^−1^ peak, corresponding to Rh6G (red: bare Au). The peak areas are calculated from spectra in a 10 × 10 μm map, with a spectrum measured every 1 μm. The addition of the SiO_2_ layer (yellow histogram) shows a clear reduction in peak area of the Rh6G, compared to the Au nanoparticles (red histogram). The green histogram is from the SHINs exposed to the Au etchant solution. (e) Top: scanning electron microscopy (SEM) image of the drop-casted SHINs before exposure to the Au etchant solution. Bottom: SEM image after the exposure of the SHINs to the Au etchant solution, clearly showing the dissolution of the Au nanoparticles.

Chemically synthesized plasmonic nanoparticles are established in the literature as an efficient tool to enhance Raman signals. As mentioned previously, these plasmonic nanoparticles are coated with a metal oxide layer for applications in catalysis to prevent side reactions. Fig. 1a shows the fabrication of these shell-isolated nanoparticles according to an existing procedure.^1^ TEM was used to determine the size of the Au nanoparticles and confirm the presence of the SiO_2_ layer (Fig. 1b). From the TEM images, the average particle size of the Au nanoparticles was determined to be 83.5 ± 7.4 nm, with a SiO_2_ coating thickness of 4.6 ± 1.2 nm. The TEM data shown in Fig. S2† also suggests a homogeneous layer of SiO_2_ around the Au nanoparticles.

The Raman scattering signal of a Rh6G probe was measured on the uncoated Au nanoparticles to confirm whether the nanoparticles were acting as Raman enhancers. The measurement was performed by scanning over a 10 × 10 μm area with a step size of 1 μm. The red graph in Fig. 1c shows a single spectrum from this 10 × 10 μm area, in which the characteristic peaks of Rh6G are visible (see Fig. S3† for the map). The peak area of the 1362 cm^−1^ peak was calculated for each pixel and is shown in the red histogram in Fig. 1d. Large variations in the signal intensity can be observed between the pixels (averaged total signal = 2.4 × 10^5^ ± 38%), indicating heterogeneity of the measured sample. The spatial variations in signal intensity could originate from several factors, including variations in the density of plasmonic nanoparticles (due to inhomogeneous deposition of SHINs *via* drop-casting) and/or variations in the amount of Rh6G at every pixel.

The yellow graph in Fig. 1c shows a single spectrum for the SiO_2_-coated Au nanoparticles. Fig. 1d also shows the intensity distribution of the SiO_2_-coated Au nanoparticles (in yellow). The averaged total intensity of the 1362 cm^−1^ band decreased significantly, while the relative variation in the signal intensity increased (averaged total area = 3.7 × 10^4^ counts ± 40%). One of the reasons contributing to the overall decrease in signal intensity can be explained by the increase in distance between the Au nanoparticles due to the presence of an additional shell.^1,31^ The shell also acts as an insulating barrier, allowing limited electric field intensity to penetrate through it. The increase in signal variation could originate from the heterogeneity of the SiO_2_ coating, leading to a variation in distances between the Au nanoparticles. The variation in SiO_2_ coating thickness, and the variation in signal intensity could therefore be detrimental for studies where spatial variations in Raman signals are of interest for applications of SHINs in catalysis.

Next to the variation in coating thickness, it is also important that the Au is not exposed to the catalytic environment through pin-holes.^1,27^ The integrity of this metal oxide shell and the absence of the pin-holes is therefore an important characteristic to study. Previous literature studies also made use of pyridine as a probe molecule, where the exposed Au would bind to the pyridine and give rise to two ring breathing modes visible in a Raman spectrum at 1008 cm^−1^ and 1036 cm^−1^ (Fig. S4†).^32^ However, to evaluate the integrity of the SiO_2_ layer, in addition to TEM measurements and pyridine tests, we also used a new proposed method for pin-hole detection: exposure of the SHINs to an Au etchant solution containing potassium iodide (KI) and iodine (I_2_).

When exposed, the Au reacts with I_2_ to form Au-I_2_, which has an increased solubility in KI, thereby dissolving and quite literally etching Au. Therefore, if there were any Au nanoparticles that were not completely insulated, they would get etched away instantly.^33^ The equation below shows how the chemical reaction proceeds:

1 Au + 2I^−^ → AuI_2_^−^ + e^−^

To test this, the SHINs were studied with Scanning Electron Microscopy (SEM) before and after the exposure to the Au etchant (Fig. 1e). It can be seen that the honeycomb-like framework of the SiO_2_ is the only remainder after the Au etching step, indicating that the Au etchant solution was able to reach the AuNPs penetrating *via* the shell, resulting in almost complete etching of the Au. These results indicate that the SiO_2_ insulating shell formed was indeed not pin-hole-free as assumed from the results obtained from TEM and the pyridine test. It should also be noted that the liquid diffusion of the Au etchant is significantly slower than gas diffusion, so it can be assumed that gas can still diffuse through a SiO_2_ layer (with pin-holes) and reach the exposed Au during a gas-phase catalytic reaction that is being studied with SHINs. Therefore, we decided that chemical synthesis was not optimal for fabricating SHINER substrates.

To confirm this, the SHINs were also tested for enhancement before and after exposure to the Au etchant. The green histogram in Fig. 1d shows the 1362 cm^−1^ peak areas of the 10 × 10 μm map for SHINs treated with Au etchant, while the yellow histogram shows the same, but before the exposure of the SHINs to the Au etchant (all maps are shown in Fig. S3†). The green histogram shows an average total area of 1.8 × 10^4^ counts ± 67%. The Raman data suggests that a portion of the SHINs is etched away (which was also confirmed from microscopy images), leading to a decrease in the average signal intensity by a factor of two compared to the non-exposed SHINs. The increased variation suggests that clusters of intact SHINs remain, while other spots do not or barely consist of Raman signal enhancers. It also indicates that the surviving SHINs had a thicker coating. This thicker coating might also be a contributing factor to the decrease in enhancement observed for the SHINs tested after the Au etch test.

It is interesting to see how the Au etchant tests in combination with scanning electron microscopy prove to be a reliable method to check the integrity of the SiO_2_ layer. The benefit of using this method over the TEM and pyridine methods is that the latter are more prone to false positives. TEM is a rather expensive and elaborate tool requiring dedicated preparation. The ease of use and limited preparation required for Au etchant tests in combination with SEM imaging might prove to be faster and more accessible in some cases. For pyridine markers, the absence of the pyridine signal could be due to the absence of Raman enhancement and/or the absence of pin-holes. The absence of pyridine signals could also be a measurement limitation of the Raman microscope. It is still unclear how many pin-holes contribute to the increase in pyridine signal for it to be a reliable method. However, even with a single pin-hole, the Au etchant can come in contact with the plasmonic nanoparticle and etch the material away, thereby being a clear indicator of the presence of pin-holes.

### 3.2 Approach 2: lithographically fabricated SERS substrates and chemically synthesized shells

**Fig. 2:**
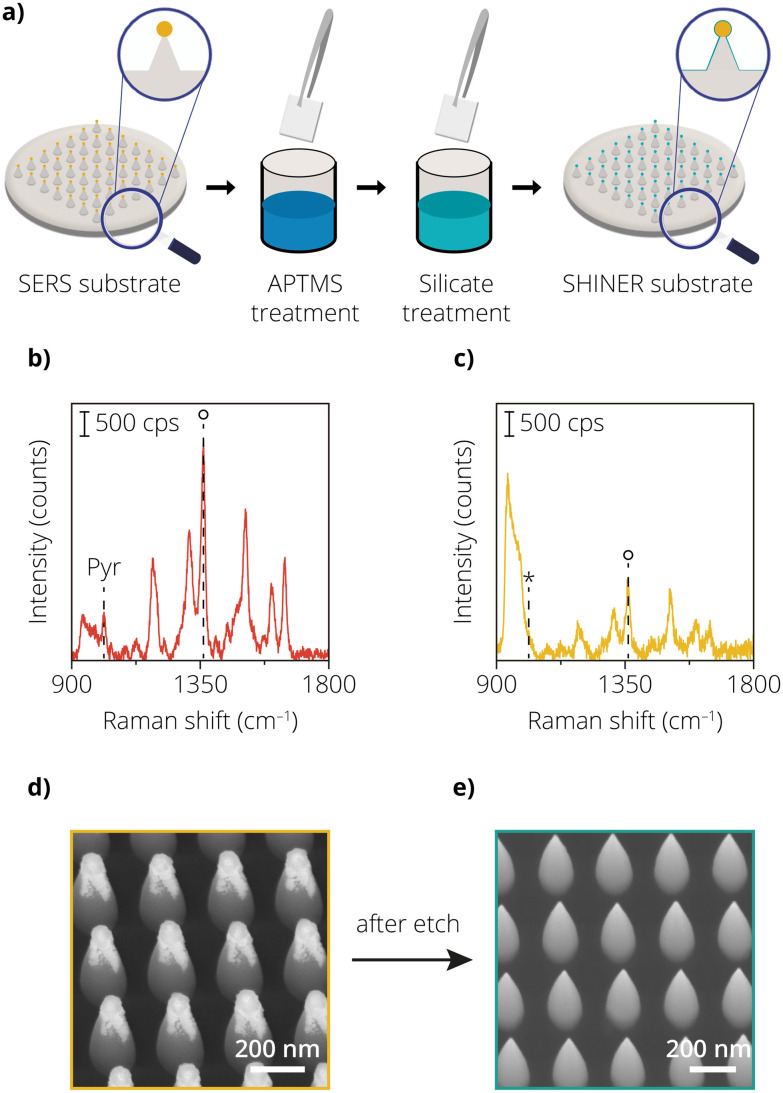
(a) Schematic overview of the preparation of the lithographically fabricated surface-enhanced Raman spectroscopy (SERS) substrates, followed by the chemically synthesized shells using APTMS/sodium silicate. (B) Raman spectrum of the lithographically fabricated sample without the SiO_2_ coating but after the addition of Rh6G and pyridine. (C) Same as in (b), but after the coating procedure. Notice that the vibration around 1000–1040 cm^−1^ is absent (shown with the asterisk), which is ascribed to the ring breathing mode of pyridine on Au. (D) Scanning electron microscopy (SEM) image of the lithographically fabricated SERS substrate with the chemically synthesized shell. (E) Same as in (d), but after the exposure to the Au etchant solution.

One of the drawbacks of using chemically synthesized nanoparticles, as highlighted in approach 1, is the high variance in the SERS signals. Upon introducing the Au etchant, the non-uniformity in the deposition method makes it difficult to differentiate between etched and non-etched nanoparticles accurately. Such an issue can be avoided with the use of lithographic nanostructures. The added advantage here is the periodicity of nanostructures. Therefore, even one missing nanostructure can be easily detected in SEM images and differentiated within a periodic arrangement offering a more quantitative analysis. Recent work showed that Au nanoparticles placed on vertically aligned silicon nanocones resulted in homogeneous enhancement factors with high orders of magnitude of ∼10^8^.^30^ A high enhancement factor ensures good quality sensing capabilities of a SERS substrate. Lithographic structures also give rise to homogeneity in the measured SERS signal, providing uniform results over a large area. In the comparative view reported in our previous work, we see that when lithographic techniques are used, SERS signal variance can be reduced to as low as 4%.^30^ For SHINERS, an additional metal oxide layer must be applied on the enhancing nanostructures to use a lithographic substrate and make it applicable for a catalytic reaction. Therefore, in approach 2, a lithographically fabricated AuNP@SiNC geometry was employed. Additionally, a SiO_2_ layer was grown using a modified chemical coating procedure schematically depicted in Fig. 2a.

To study the enhancement of the AuNP@SiNCs before and after metal oxide shell coating, the Raman spectrum of Rh6G was measured. Fig. 2b shows the Raman spectrum of Rh6G on the uncoated sample. The peak area of the 1362 cm^−1^ peak is lower than that of the AuNPs in approach 1. We ascribe this difference to a lower amount of plasmonic nanoparticles in the probed area (1 μm^2^), caused by the larger spacing between the nanoparticles on top of the nanocones. The nanocones are spaced in a 2D array with a pitch of 250 nm between the AuNPs. This highlights one of the limitations of lithography compared to chemically synthesized nanoparticles. While with chemical synthesis routes, metal nanoparticles can be placed in close proximity to each other (∼2–10 nm),^34^ it is quite difficult to do so with lithographic tools. Recent literature reviews also show that the minimum spacing that can be achieved with lithographic tools is typically in the range of a few hundred nanometers.^35–39^ It should be noted that SERS substrates based on chemically synthesized metal nanoparticles typically rely on near-field coupling due to their close proximity to each other.^40,41^ This contributes to higher SERS signals compared to the far-field coupled lithographic nanostructures. Typically, optical tools fabricating SERS substrates are limited by either the diffraction limit of light or the proximity effects observed when using electron beams for exposure.^42^ This limits the physical proximity that two enhancing nanostructures can achieve. This difference can also be evidenced by the ratio of SERS intensity of Rh6G between approach 1 and 2 where in approach 1 the SERS substrate consists of near-field coupled plasmonic structures while in approach 2 the SERS substrate consists of far-field coupled plasmonic nanostructures.

Fig. 2c shows the Raman spectrum of the chemically synthesized shell-coated nanoparticles on the nanocones, using Rh6G as a probe molecule. The presence of Rh6G can be confirmed by observing the characteristic peaks of Rh6G at 1305 cm^−1^, 1362 cm^−1^ and 1512 cm^−1^ for both the shell-isolated (coated) and uncoated samples.^43^ Additionally, to detect the presence of pin-holes, the samples were also treated with pyridine. Since the uncoated samples have not been subjected to any process steps and have no insulating shell, the characteristic peaks of pyridine at 1008 cm^−1^ and 1036 cm^−1^ are observed.^12^ Compared to the SHINs from the first approach, the decrease in the 1362 cm^−1^ peak area for the coated nanoparticles on the nanocones is only a factor 2.5 (from 1.0 × 10^5^ to 4.0 × 10^4^), compared to a drop in peak area by a factor 6 for the SHINs in approach 1, after the Au nanoparticles were insulated (Fig. 1d). We estimate that the added thickness of the SiO_2_ coating has a smaller contribution to the total spacing between the nanoparticles and, therefore, a smaller effect on the drop in enhancement. Despite the presence of the SiO_2_ insulating layer, we clearly see that the electric field enhancement is able to penetrate through the shell, making it possible to detect Rh6G. The presence of a SiO_2_ shell can also be witnessed by the increase in the second-order peak of Si at 980 cm^−1^.^44^ For the coated sample, the pyridine test again shows an apparent absence of pin-holes since there are no visible vibrations in the 1000–1050 cm^−1^ region that can be ascribed to the ring breathing modes of pyridine.^32^

The Au etchant test however proved differently as shown by the SEM images before (Fig. 2d) and after (Fig. 2e) the exposure of the coated sample to the Au etching agent. Before the etching test, the Au nanoparticles present on the tips of the nanocones could also withstand harsh treatments such as exposure to liquids (*e.g.*, rinsing with DI water, HNO_3_). However, when subjected to Au etchant, not only did the AuNP not survive, but there was also no residual insulating shell around the nanocone geometry. We hypothesize that the insulating shell that was chemically synthesized had multiple pin-holes and, therefore, multiple points of entry for the Au etchant to dissolve the AuNP and also remove the shell. This also causes the shell to break off, when the structural support of the AuNP at the tip is missing. Based on these experiments, we assess that the chemical coating procedure resulted in SiO_2_ coatings that are not resistant to the Au etchant, confirming the presence of pin-holes. These experiments also highlight the limitations of using pyridine as a probe molecule to detect pin-holes. We therefore suggest the use of an Au etchant test as an additional method to check for the presence of pin-holes.

### 3.3 Approach 3: lithographically fabricated SERS substrate and ALD shells

**Fig. 3:**
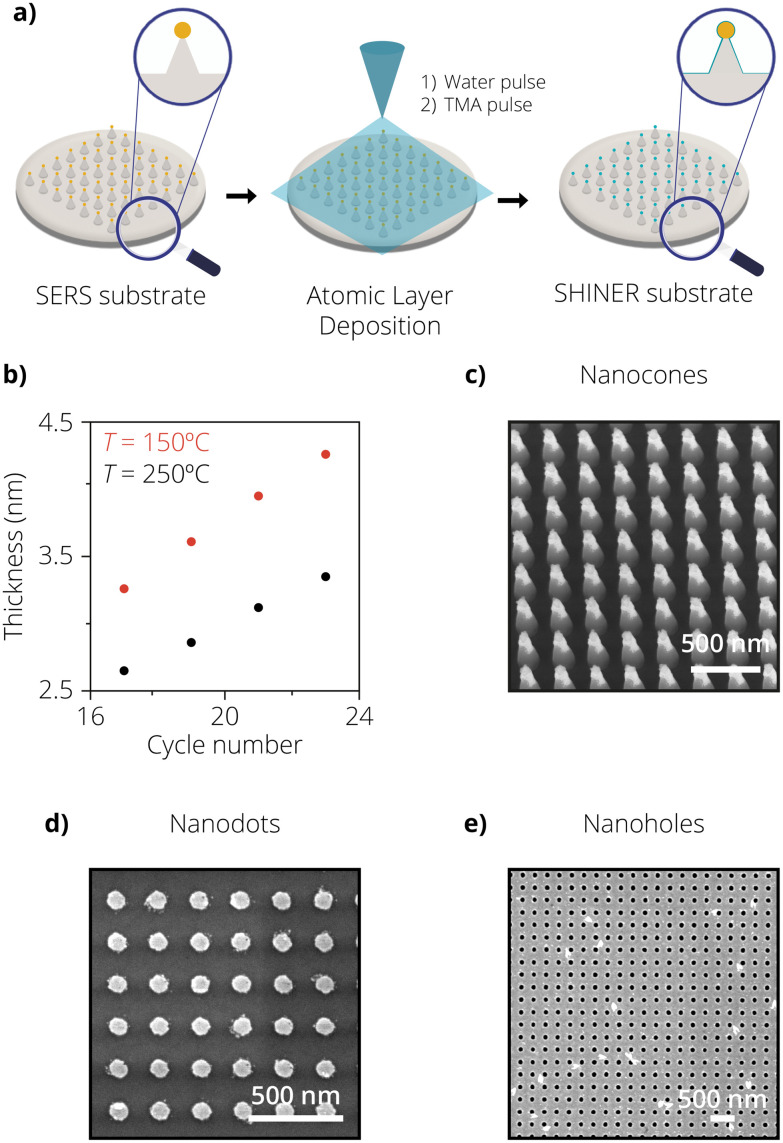
(a) Schematic overview of the preparation of the lithographically fabricated SERS substrates, followed by atomic layer deposition (ALD). (b) Plot showing the layer thickness, as measured by ellipsometry measurements. The ALD samples were measured after different cycle numbers and different process temperatures, 150 °C (red) and 250 °C (black), respectively. (c) SEM image of the lithographically fabricated nanocones. (d) SEM image of the lithographically fabricated nanodots. (e) Scanning electron microscopy (SEM) image of the lithographically fabricated nanoholes.

In our third approach, both the SERS substrate and the insulating shell were fabricated using lithographic tools to evaluate the performance of a fully lithographic SHINER substrate. In this approach, the combination of electron beam lithography (EBL), displacement talbot lithography (DTL), metal deposition followed by lift-off, and ALD was used to fabricate two different geometries, nanodots and nanoholes. ALD was chosen as a method to replace the chemical synthesis routes for shell isolation, while optical lithographic tools were used for the SERS substrate fabrication. Fig. 3a shows a graphical representation of the process flow followed. The work of T. Wu *et al.*^45^ inspired the design of the SERS substrate, which consists of 90 nm Au dots arranged in a square periodic lattice. The geometry was chosen for its simplicity and ease of fabrication.

#### 3.3.1 Atomic layer deposition optimization

When a plasmonic nanoparticle core is coated with a shell, its optical properties can be modified significantly due to the distinct change in the dielectric constant of the system. Commonly used materials for forming pin-hole-free shells include SiO_2_, Al_2_O_3_ and silicon nitride (SiN_*x*_).^46,47^ It has been experimentally shown before that a shell with a higher refractive index leads to a stronger SERS signal.^46^ This is primarily because as the refractive index increases, the real part of the effective refractive index needs to be more negative in order to satisfy the resonance condition. This leads to a shift in resonance frequency which is observed as a red-shift in the localized surface plasmon resonance. Since light needs to bend more when entering a material with a higher refractive index, it results in a much higher enhancement in the localized electric field.^48^ In this section, the deposition of Al_2_O_3_ instead of SiO_2_ was chosen because of the technical restrictions of the used ALD system and the availability of the TMA precursor. However, since Al_2_O_3_ has a higher refractive index than SiO_2_, this would also result in a better SERS enhancement when compared to using a SiO_2_ shell. The chemically synthesized shell was made from SiO_2_ in approaches 1 and 2, as the requirements for the shell (*i.e.*, ultrathin and durable) have not yet been met for a chemically synthesized shell of Al_2_O_3_. We have only found ALD as a method to fabricate the Al_2_O_3_-based shells around the plasmonic nanostructures.^18,46,49,50^

One of the greatest advantages of ALD is the precise control of the deposited coating thickness. Each deposition consists of (at least) two subsequent precursor pulses, forming a monolayer of the deposited material. Each cycle therefore deposits a certain thickness (∼0.147 nm at 250 °C in our case, for TMA and water) of the resulting material onto a substrate. The total coating thickness can be tuned by varying the cycle number. Fig. 3b shows a linear trend in the film thickness of Al_2_O_3_ for increasing cycle numbers. To determine the thickness of the film, bare silicon substrates were coated as dummies with different deposition cycle numbers. More details on the mechanism of the ALD shell formation can be found in the work of Ghosh *et al.*^51^ Optical models with ellipsometry measurements were then used to determine the thickness and the homogeneity of the films, which can also be seen in Fig. 3b. Fig. S5† shows the cross-sectional view of an ALD coated Au–Si substrate with a 10 nm Al_2_O_3_ shell revealing its smoothness and pin-hole-free nature. Energy-dispersive X-ray (EDX) mapping was additionally performed on the samples to confirm the presence of the aluminum, indicating that in fact, an Al_2_O_3_ shell had been deposited on the samples. This is shown in Fig. S6.† Fig. S6(b) and S5(c)† show that the Al_2_O_3_ layer not only deposits on the silicon substrate but also on the Au reference area. The images and EDX analysis were performed after the samples were exposed to the Au etchant test. In addition to the cycle numbers, it can also be seen that the process temperature influences the film thickness as well. For a higher process temperature, a more crystalline coating is deposited. At a given number of deposition cycles, the more crystalline coating formed at higher temperatures (250 °C) leads to a thinner and more dense coating compared to the more amorphous and less dense one at lower temperatures (150 °C). This can be evidenced by the plot in Fig. 3b.^52^

To test the durability of the shells and confirm their insulating nature, samples consisting of Au reference areas were subjected to various cycles of Al_2_O_3_ deposition to form insulating shells of different thicknesses. The samples were then submerged in Au etchant for 30s and analysed afterward with SEM. The results of these experiments can be visualized in Fig. S7.† It can be seen for 17 deposition cycles that the residues of a patchy Au film can be observed. This occurs due to pin-holes in the insulating shell, allowing Au etchant to seep through and etch the underlying Au film. In the SEM image, the lighter-coloured areas represent the Au film, while the darker ones represent the underlying Si substrate. However, this is not the case for 19 deposition cycles, where no such patches can be observed until the sample edge, which reveals that a perfectly homogeneous insulating shell has been formed. These results determined that the best conditions for forming a pin-hole-free insulating shell for a non-patterned Au film was 19 deposition cycles at 250 °C yielding an Al_2_O_3_ shell thickness of 2.8 nm.

The same experiments were also repeated for the AuNP@SiNC, nanodots, and nanohole geometries. Each sample was coated with an increased shell thickness until all the nanostructures survived the Au etch test. The SEM images shown in Fig. 3c and d consisting of AuNP@SiNC, Au nanodots, and Au nanoholes were taken after the samples were dipped in Au etchant for 30 s and rinsed with DI water. It can be seen that the nanostructures survived the Au etch test after 23 (cones), 19 (dots), and 23 (holes) deposition cycles, respectively. This means that the nanodots were isolated with a 2.8 nm thick Al_2_O_3_ shell, while the nanoholes and AuNP@SiNCs were isolated with a 3.1 nm thick Al_2_O_3_ shell.

It is interesting to see how different geometries change the number of cycles needed to form an insulating shell. We hypothesize that the increasing number of cycles needed to form an insulating shell on nanoholes and AuNP@SiNCs compared to nanodots could be attributed to surface roughness. It has been reported that the first few cycles of ALD deposition result in a more island-like deposition instead of a continuous film.^28^ Therefore, the surface roughness of the samples can be a critical aspect influencing the number of cycles needed to form a closed pin-hole-free shell, especially given the range we are interested in (2–3 nm). In the case of Au nanoholes, during the lift-off process, residual Au flakes redeposit on the Au nanohole surface. This contributes to additional surface roughness, which Au nanodots do not possess due to the ease in fabrication of the lift-off process. This can also be visualized by the SEM images of the Au nanodots and Au nanoholes, where the redeposited Au flakes can clearly be seen for Au nanoholes. Fig. S8† shows the SEM image of AuNP on SiNCs after 19 deposition cycles. After an Au etch test, it can be clearly seen that some of the AuNPs are missing as they have been etched away due to the presence of pin-holes. If looked closely, one can also observe the hollow Al_2_O_3_ shell present where the AuNP should have been located.

**Fig. 4:**
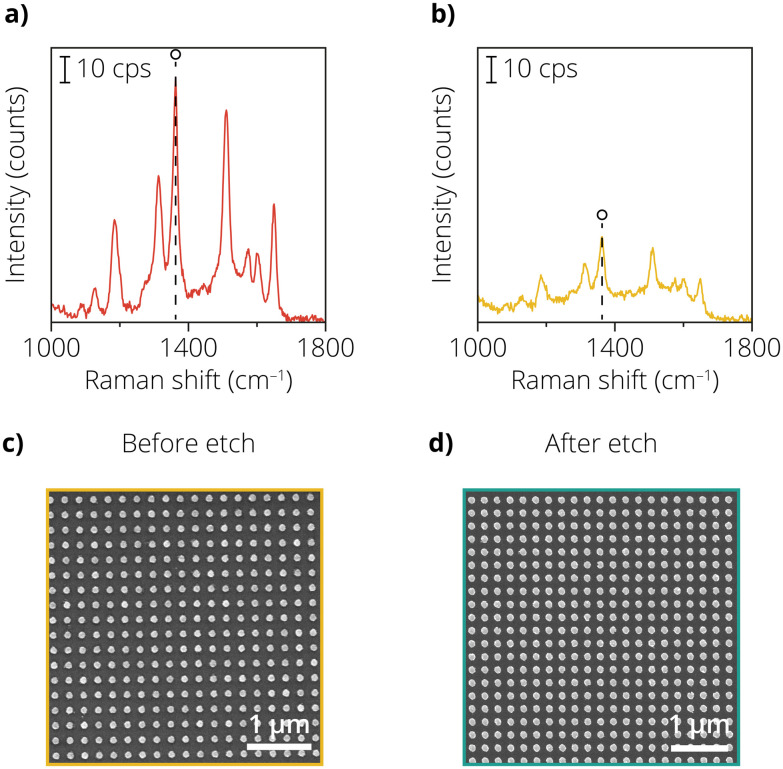
(a) Raman spectrum of the lithographically fabricated nanodots with Rh6G. (b) Same as in (a), but after the atomic layer deposition (ALD) procedure creating the shell around the Au. (c) Scanning electron microscopy (SEM) image of the coated nanodots. (d) Same as in (c), but after the exposure to the Au etchant solution.

It can be seen from previous work^1^ that for shell thicknesses greater than 3 nm, the SERS enhancement is very limited and not sufficient to measure catalytic reactions at low laser powers. The Au etchant experiments determined that the Au nanodots were the optimal geometry to form lithographic SHINER substrates with conformal and pin-hole-free insulating shells. This was done as Au nanodots required the least amount of cycles to form a completely insulating shell. Fig. 4a and b show the Raman enhancement study for nanodots both with and without the deposited shell. Rh6G was used as a probe molecule, and the peak area of the 1362 cm^−1^ band was calculated. Fig. 4a shows the Rh6G spectrum for the nanodots without an ALD layer. The nanodots showed an average peak area of 1.9 × 10^4^ ± 19% (maps are shown in Fig. S9†). Note that the variability of the signal is drastically lower than with the other SERS substrates, indicating that the nanodots are a more homogeneous substrate. The average peak area is comparable to the nanocones, indicating that the amount of plasmonic material in the measured area is a descriptor for the amount of enhancement.

Fig. 4b shows that introducing a 2.8 nm thick ALD Al_2_O_3_ layer led to a reduction in the peak area of the 1362 cm^−1^ band. The signal intensity decrease was around a factor of three again, comparable to the nanocones in approach 2. The average peak area was 7.5 × 10^3^ ± 17%. The low variability in the signal indicates that the samples provide relatively homogeneous enhancement of the Rh6G signal. However, the absolute enhancement is significantly lower than the SHINs, due to the difference in the type of enhancement: the SHINs exhibit near-field enhancement, the nanodots show far-field enhancement due to the periodic lattice and larger distance between the particles. Fig. 4c and d show the SEM images before and after the Au etch test on the nanodots with the ALD-prepared layer. The particles remain intact, indicating that the ALD layer successfully protects the Au nanoparticle. The spectra for Rh6G on the Al_2_O_3_ coated nanodots were taken after subjecting the sample to the Au etch test and visualizing in SEM to ensure that the sample was pin-hole-free.

This approach shows the powerful combination of lithography and ALD to fabricate reproducible SHINER substrates. With this combination, we can alleviate problems such as homogeneity and SERS signal variance while fabricating pin-hole-free insulating shells. We further confirm that the formed shells are pin-hole-free by using an Au etchant method, which has proven to be a reliable technique for checking the durability of the insulating shells.

## 4. Conclusions

We provide a method to fabricate reliable and reproducible SHINER substrates, demonstrating a promising potential for the field of catalysis. We show a comparative view between lithographic tools and chemical synthesis routes for fabricating the SERS substrate base and the insulating shell. While chemical synthesis routes offer extremely high enhancing capabilities due to the near-field coupling of the nanostructures, they often lack reproducibility and homogeneity, which limits their widespread application in catalytic reaction sensing. Additionally, chemically synthesized shells have also been proven to contain pin-holes, which were detected by introducing an Au etchant test. To overcome these issues, we use ALD as an alternative method for fabricating insulating shells that are durable, conformal, and reproducible. The Au etchant test was used to confirm that the shells fabricated using ALD were pin-hole-free. We show that Au nanodots can be a potential tool for studies where homogeneous enhancement is required while ensuring no ‘contamination’ from the Au by using insulating shells. However, it should be noted that for many applications, the SERS signals obtained from such a geometry are still too low due to the far-field limitations in the enhancement. Further investigation and combination of higher enhancing substrates with ALD-coated layers is needed to achieve a better signal-to-noise ratio.

## Author contributions

Ketki Srivastava: conceptualization, data curation, formal analysis, investigation, methodology, software, validation, visualization, project administration, writing – original draft, writing – review and editing; Thimo S. Jacobs: conceptualization, data curation, formal analysis, investigation, methodology, software, validation, visualization, project administration, writing – original draft, writing – review and editing; Stefan Ostendorp: resources, validation, writing – review and editing; Dirk Jonker: conceptualization, resources, writing – review and editing; Floor A. Brzesowsky: conceptualization, resources; Arturo Susarrey-Arce: conceptualization, resources, writing – review and editing; Han Gardeniers: resources, writing – review and editing; Gerhard Wilde: resources, writing – review and editing; Bert M. Weckhuysen: resources, funding acquisition, validation, visualization, supervision, project administration; Albert van den Berg: resources, funding acquisition, validation, visualization, supervision, project administration; Ward van der Stam: conceptualization, resources, validation, visualization, supervision, project administration, writing – review and editing; Mathieu Odijk: conceptualization, resources, validation, visualization, supervision, project administration, writing – review and editing.

## Conflicts of interest

There are no conflicts to declare.

## Supplementary Material

NR-016-D4NR00428K-s001
