# Supplementary material for: Alternative nano-lithographic tools for shell-isolated nanoparticle enhanced Raman spectroscopy substrates
Source: Nanoscale. 2024 Mar 14;16(15):7582–93. doi: 10.1039/d4nr00428k (PMC11025715; doi:10.1039/d4nr00428k)
Supplement: NR-016-D4NR00428K-s001 [file NR-016-D4NR00428K-s001.pdf]

Electronic Supplementary Material (ESI) for Nanoscale.
This journal is © The Royal Society of Chemistry 2024

Alternative Nano-lithographic Tools for Shell-Isolated Nanoparticle Enhanced Raman Substrates

Ketki Srivastava[a†], Thimo S. Jacobs[b†], Stefan Ostendorp[c], Dirk Jonker[d], Floor A. Brzesowsky[b], Arturo Susarrey Arce[d], Han Gardeniers[d], Gerhard Wilde[c], Bert M. Weckhuysen[b], Albert van den Berg[a], Ward van der Stam[b], Mathieu Odijk[a]

Supplementary Information

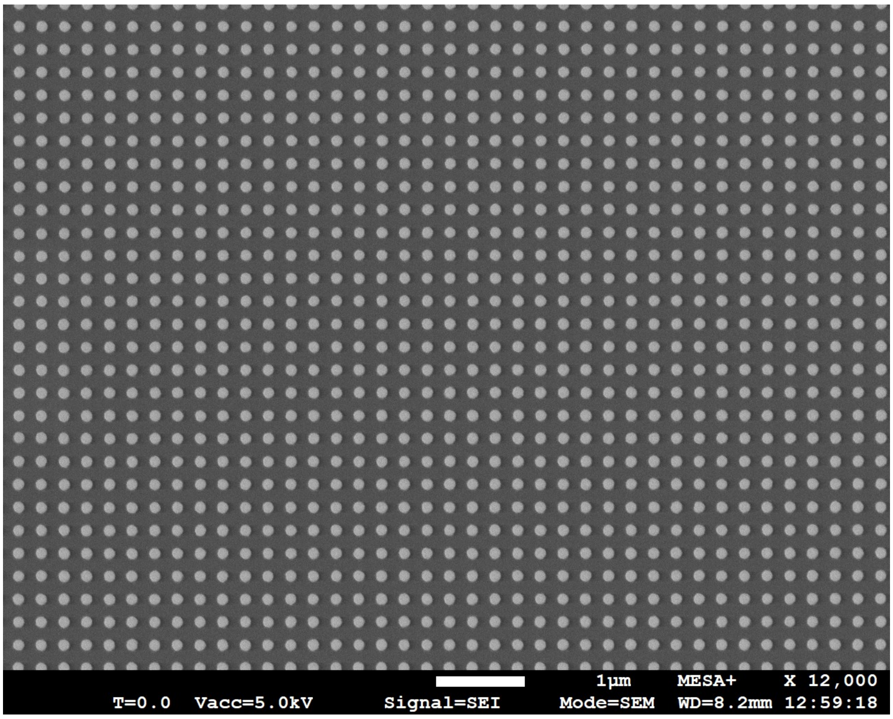

Figure S1: BARC+PFI-88 nanodots fabricated on a silicon wafer by displacement talbot lithography

Table S1:Process flow followed for the deposition of 1 layer of Al_2O_3

Step No.	Step name	Parameter value	Purpose
1.	Flow	20 sccm	increased gas flow helps cleaning the system from precursor residues
2.	Wait	10 s	time for cleaning at increased gas flow
3.	Flow	10 sccm	reduced gas flow to set process pressure and improves precursor retention time

4.	Wait	2 s	time to stabilize at process pressure
5.	Stopvalve	0	closes vacuum supply, causes increased precursor retention
6.	Pulse	0.015 s	H_2O vapor pulse, time adjusts partial pressure of the precursor
7.	Wait	5 s	retention time
8.	Stopvalve	1	opens vacuum supply
9.	Flow	20 sccm	increased gas flow helps cleaning the system from precursor residues
10.	Wait	8 s	time for cleaning at increased gas flow
11.	Flow	5 sccm	reduced gas flow to set process pressure and to improve precursor retention time
12.	Wait	2 s	time to stabilize at process pressure
13.	Stopvalve	0	closes vacuum supply, causes increased precursor retention
14.	Pulse	0.015 s	TMA vapor pulse, time adjusts partial pressure of the precursor
15.	Wait	5 s	retention time
16.	Stopvalve	1	opens vacuum supply

The thickness of the coating can be adjusted by changing the cycle number which repeats the steps from step 1 to step 16. It is important to note that after each pulse, a wait step of 5 seconds is added as a hold time, during which the vacuum system is shut off, the continuing gas flow increases the pressure in the system, but the precursor remains for longer at the sample surface. This ensures that the precursor material has enough time to cover all the surface of the nanostructures. This modification is typically used for high aspect ratio structures to ensure conformal deposition of the material. 15 ms pulse time was chosen based on the characteristics of the machine in terms of reaction

volume, gas flux and the necessary reaction pressures. The rather short pulse time is still long enough to ensure that there is no lack of precursor material during the deposition process. The subsequent N_2 purging step was used to clean the chamber and remove excess precursor material.

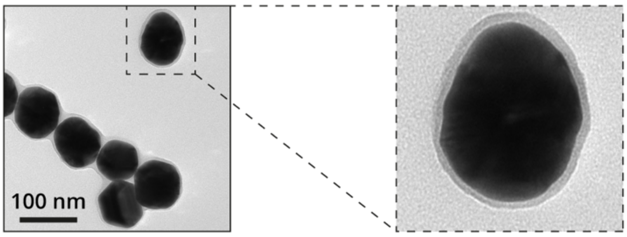

Figure S2: TEM image of the SHINs with a zoom-in on an individual nanoparticle, showing the SiO_2 coating around the particle. The scalebar in the image is 100 nanometer.

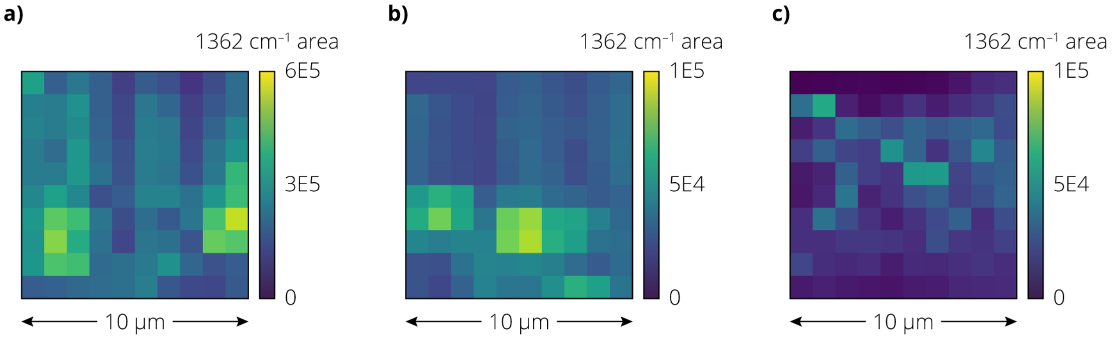

Figure S3: Two-dimensional (2D) maps of the 1362 cm^{-1} peak areas of Rh6G on (a) gold nanoparticles, (b) SHINs and (c) SHINs after etching, respectively.

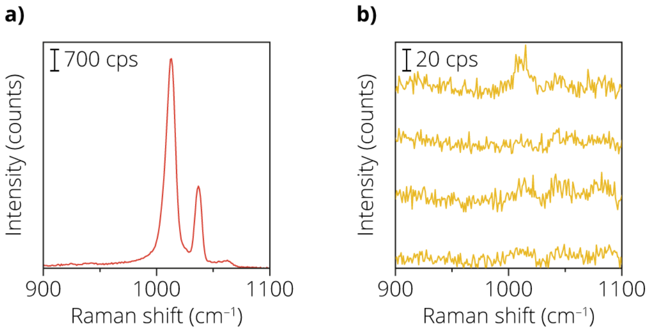

Figure S4: a) Raman spectrum of the pyridine adsorbed on the gold nanoparticles. Two peaks at 1008 and 1030 cm^{-1} can be observed. b) Same as in a), but now for the chemically synthesized shells on the gold nanoparticles. Four spectra are plotted with an offset, to show the variance of the signal and the difficulty to observe or exclude the pyridine vibration.

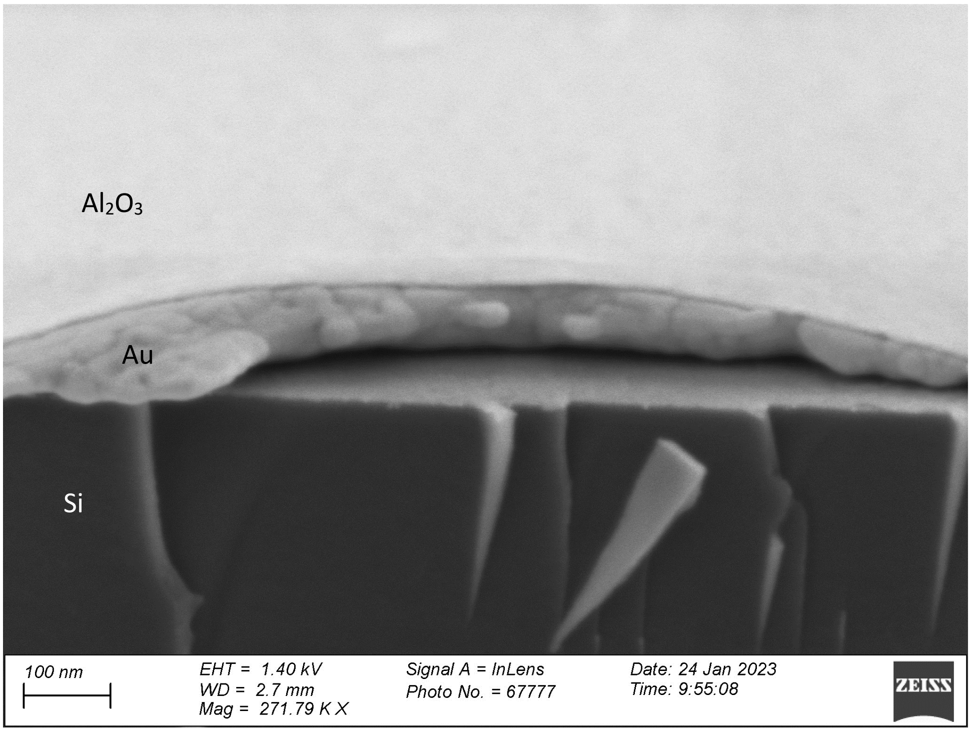

Figure S5: Cross-sectional view of an aluminium oxide (Al_2O_3) coated gold (Au) silicon substrate. AL_2O_3 (t) = 10 nm, Au (t) = 50 nm

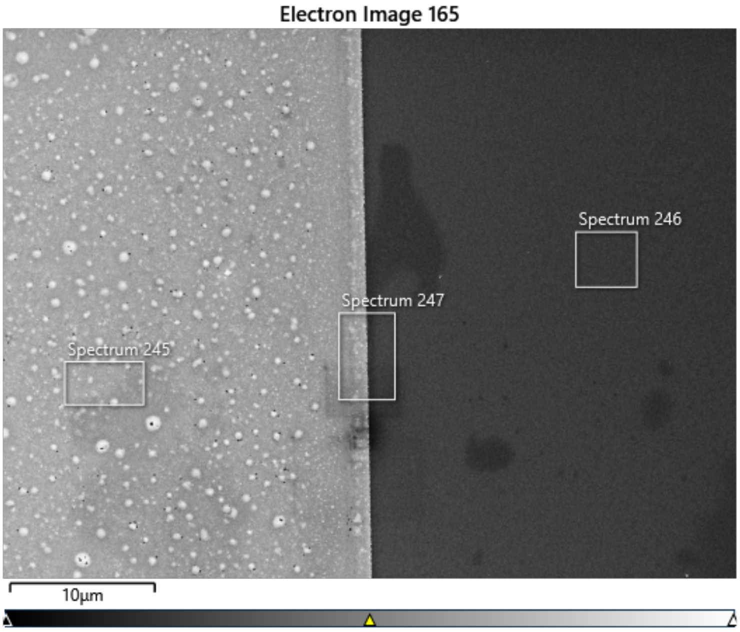

Figure S6 (a): High-resolution scanning electron microscopy image of a 1 mm^2 gold square (left side) on a silicon substrate.

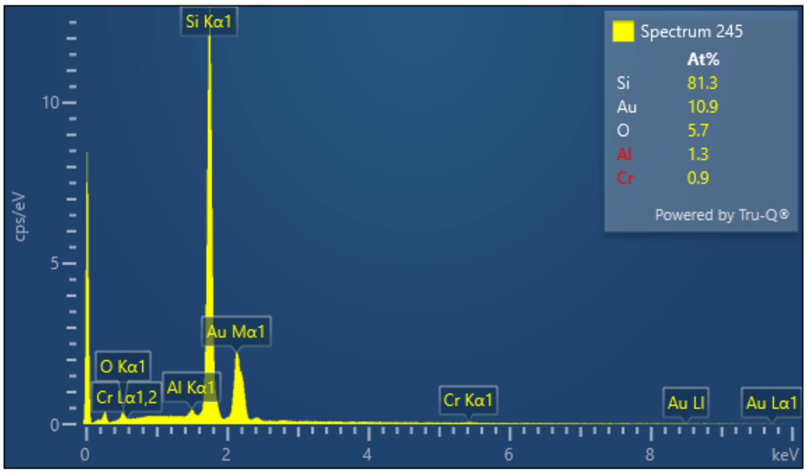

Figure S6 (b): Energy-dispersive X-ray spectroscopy analysis of spectrum 245 (indicated in figure S5(a).

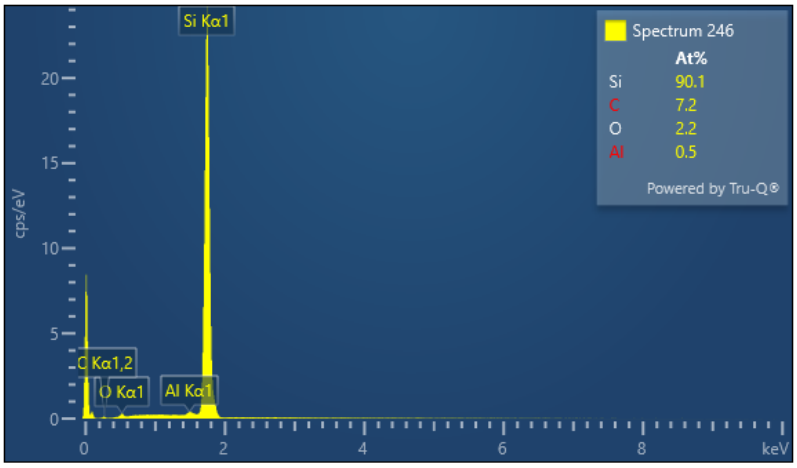

Figure S6 (c): Energy-dispersive X-ray spectroscopy analysis of spectrum 246 (indicated in figure S5(a).

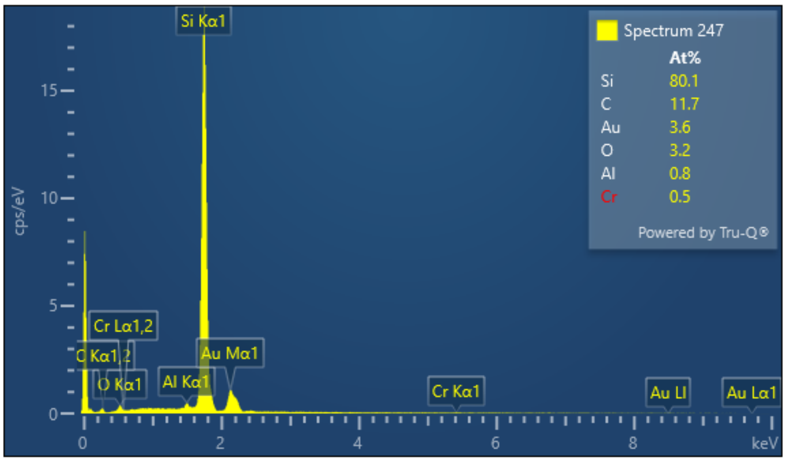

Figure S6 (d): Energy-dispersive X-ray spectroscopy analysis of spectrum 247 (indicated in figure S5(a).

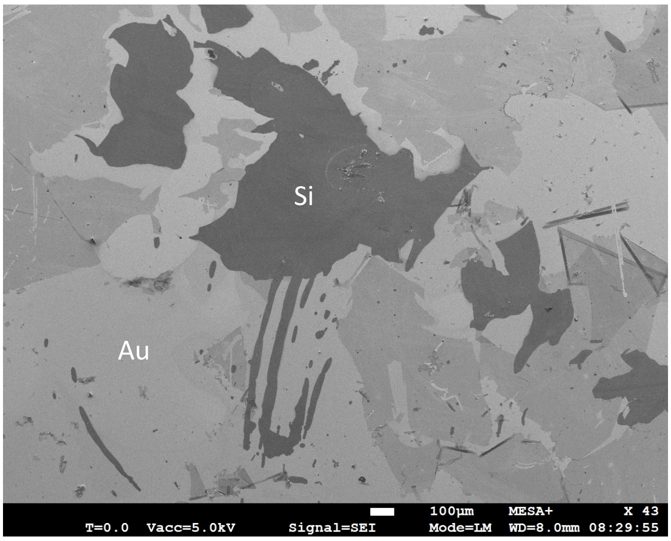

Figure S7 (a): Gold reference sample with 17 ALD deposition cycles of Al_2O_3 subjected to gold etchant test.

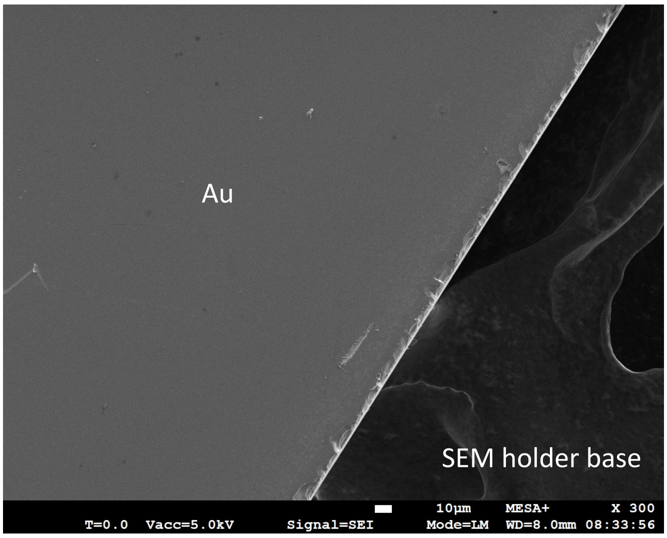

Figure S7 (b): Gold reference sample with 19 ALD deposition cycles of Al_2O_3 subjected to gold etchant test

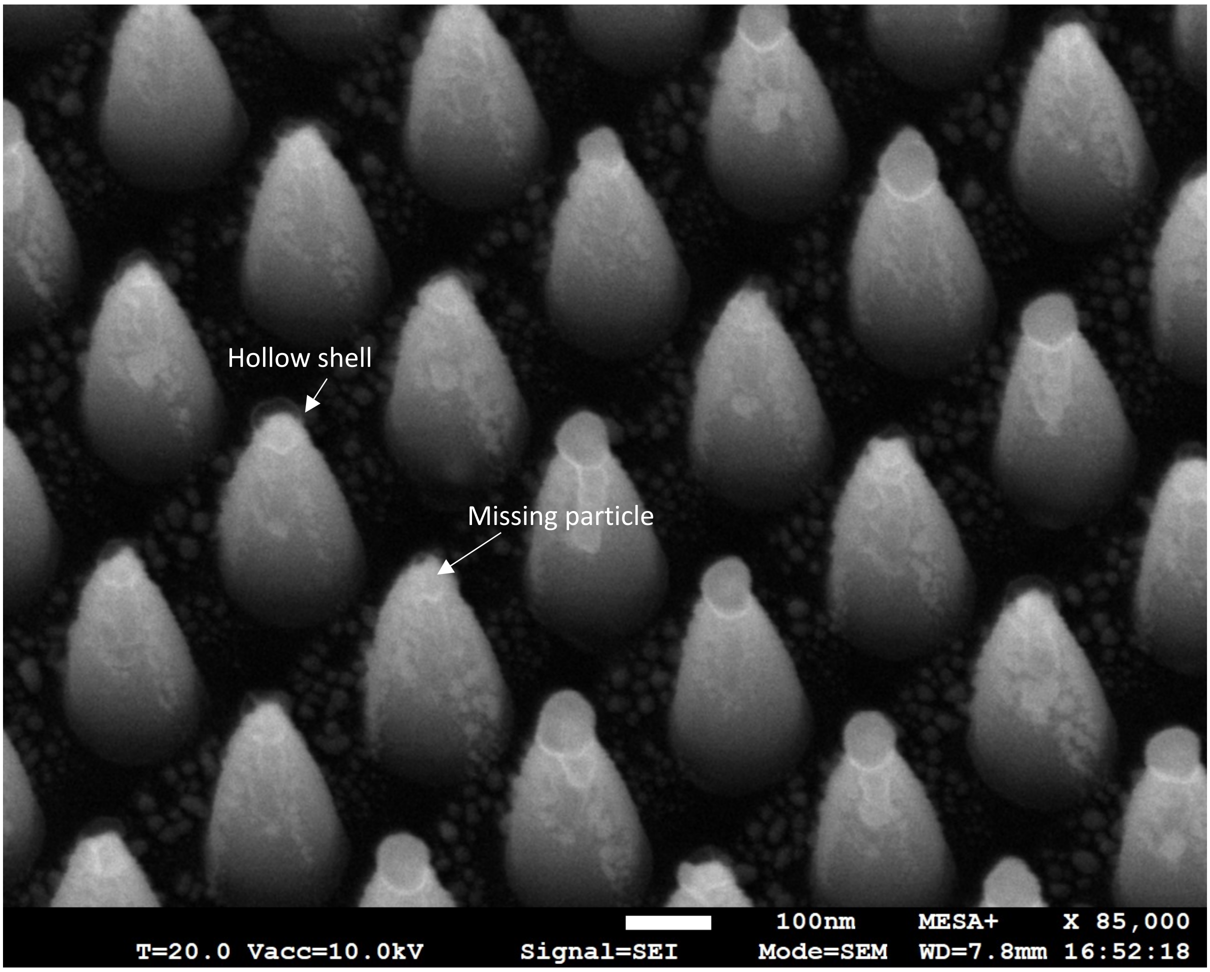

Figure S8: AuNP@SiNC coated with 19 ALD deposition cycles of Al_2O_3. Missing gold nanoparticles can be visualized on top of the nanocones

Table S2: Overview of the (average) signal area of the 1362 cm^{-1} peak with the respective variance over the measured 100 pixels of 1 μm^2

Sample	Average signal (100 pixels)	Variance in signal
AuNPs + Rh6G	241456	38.2%
AuNPs@SiO_2 + Rh6G	37045	40.4%
AuNPs@SiO_2 + Rh6G after etching	18270	67.2%
AuNCs	101727	Single spot
AuNCs@SiO_2	40317	Single spot
Au Nanodots	18800	18.6%
Au Nanodots@Al_2O_3	7467	17.2%

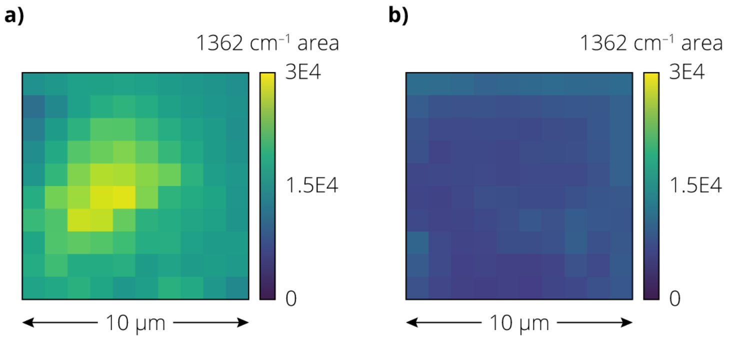

Figure S9: 2D maps of the 1362 cm^{-1} peak area of Rh6G on (a) nanodots and (b) nanodots with Al_2O_3 shell, respectively.

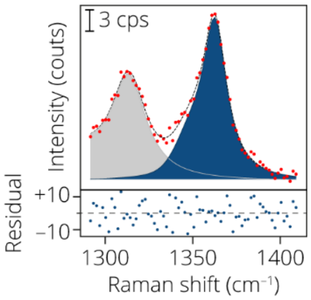

Figure S10: Voigt peak fitting of the 1362 cm^{-1} peak from the Raman spectrum depicted in Figure 4b. The area between 1300 and 1400 cm^{-1} was used, with two different Voigt line shapes for the two distinct Rh6G vibrations.
